# Hypoxic erythrocytes mediate cardioprotection through activation of soluble guanylate cyclase and release of cyclic GMP

**DOI:** 10.1172/JCI167693

**Published:** 2023-09-01

**Authors:** Jiangning Yang, Michaela L. Sundqvist, Xiaowei Zheng, Tong Jiao, Aida Collado, Yahor Tratsiakovich, Ali Mahdi, John Tengbom, Evanthia Mergia, Sergiu-Bogdan Catrina, Zhichao Zhou, Mattias Carlström, Takaaki Akaike, Miriam M. Cortese-Krott, Eddie Weitzberg, Jon O. Lundberg, John Pernow

**Affiliations:** 1 Department of Medicine, Unit of Cardiology, Karolinska Institutet, Stockholm, Sweden.; 2 Department of Physiology, Nutrition and Biomechanics, The Swedish School of Sport and Health Sciences, Stockholm, Sweden.; 3 Department of Molecular Medicine and Surgery, Karolinska Institutet, Stockholm, Sweden.; 4 Institute for Pharmacology and Toxicology, Ruhr-University Bochum, Bochum, Germany.; 5 Department of Physiology and Pharmacology, Karolinska Institutet, Stockholm, Sweden.; 6 Department of Environmental Medicine and Molecular Toxicology, Tohoku University Graduate School of Medicine, Sendai, Japan.; 7 Myocardial Infarction Laboratory, Division of Cardiology, Pneumology and Vascular Medicine, Medical Faculty, Heinrich-Heine-University, Düsseldorf, Germany.; 8 Department of Cardiology, Karolinska University Hospital, Stockholm, Sweden.

**Keywords:** Cardiology, Hematology, Cardiovascular disease, Hypoxia, Nitric oxide

## Abstract

Red blood cells (RBCs) mediate cardioprotection via nitric oxide–like bioactivity, but the signaling and the identity of any mediator released by the RBCs remains unknown. We investigated whether RBCs exposed to hypoxia release a cardioprotective mediator and explored the nature of this mediator. Perfusion of isolated hearts subjected to ischemia-reperfusion with extracellular supernatant from mouse RBCs exposed to hypoxia resulted in improved postischemic cardiac function and reduced infarct size. Hypoxia increased extracellular export of cyclic guanosine monophosphate (cGMP) from mouse RBCs, and exogenous cGMP mimicked the cardioprotection induced by the supernatant. The protection induced by hypoxic RBCs was dependent on RBC-soluble guanylate cyclase and cGMP transport and was sensitive to phosphodiesterase 5 and activated cardiomyocyte protein kinase G. Oral administration of nitrate to mice to increase nitric oxide bioactivity further enhanced the cardioprotective effect of hypoxic RBCs. In a placebo-controlled clinical trial, a clear cardioprotective, soluble guanylate cyclase–dependent effect was induced by RBCs collected from patients randomized to 5 weeks nitrate-rich diet. It is concluded that RBCs generate and export cGMP as a response to hypoxia, mediating cardioprotection via a paracrine effect. This effect can be further augmented by a simple dietary intervention, suggesting preventive and therapeutic opportunities in ischemic heart disease.

## Graphical abstract

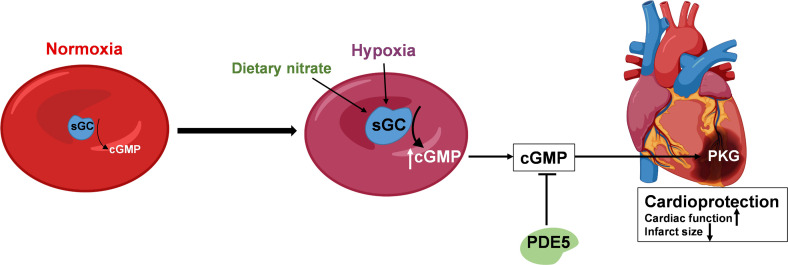

## Introduction

There are several lines of evidence suggesting that red blood cells (RBCs), in addition to their fundamental role as transporters of oxygen, are involved in the regulation of cardiovascular homeostasis. It was suggested early that the RBCs are capable of inducing a vasodilation, especially under hypoxic conditions orchestrated by the generation and export of a vasodilator signal upon deoxygenation of hemoglobin (1–3). By sensing oxygen, RBCs thereby have the unique ability to regulate cardiovascular function in situations of hypoxia. The identity of this signal has been proposed to be nitric oxide (NO) or adenosine triphosphate (ATP) (4–8) but this still remains controversial (2, 6, 9–11). The ability of the RBC to export NO bioactivity has been a matter of substantial discussion, particularly because of the ultrarapid and effective scavenging of NO by hemoglobin (11).

The controversy behind the formation and export of NO in RBCs has led to a remaining skepticism regarding a functional role of RBCs as a transducer of NO bioactivity. It has long been understood that RBCs express a functional endothelial NO synthase (eNOS) (7, 12). Further evidence supporting an important role of RBC-derived NO bioactivity came from a study on isolated hearts exposed to ischemia reperfusion (IR). It was demonstrated that RBCs exported a cardioprotective signal that was dependent on the presence of eNOS in the RBC (13). This action of the RBC was recently confirmed to exist also under in vivo conditions (14). These findings emphasize the important role of the RBC as a mediator of cardiovascular protection in hypoxic/ischemic conditions. The signaling downstream of eNOS and NO formation in the RBC remains unknown, however. It was recently demonstrated that the RBC also carries soluble guanylate cyclase (sGC), which forms cGMP, leading to activation of protein kinase G (PKG) (15). This indicates that the RBC has the capability to signal the complete NO-sGC-cGMP-PKG pathway. However, it remains unclear to what extent this signaling pathway is activated in the RBC and whether this occurs during hypoxia to protect from cardiac IR injury. Thus, there is a clear need for determining whether RBCs exposed to hypoxia have the capability to protect the heart during IR and for the identification of the mediator formed by the NO signaling pathway and exported from hypoxic RBCs to induce cardioprotection.

The present study was therefore undertaken with the purpose of investigating in detail the NO signaling pathway in the RBC exposed to hypoxia and how this pathway mediates cardiac protection. Using a well-documented model of cardiac IR (13, 16), we demonstrate that RBCs exposed to hypoxia export cyclic guanosine monophosphate (cGMP) via a mechanism dependent on the cGMP-producing enzyme sGC within the RBC to induce cardioprotection via a paracrine signaling. This cardioprotective effect is augmented by inorganic nitrate to increase sGC signaling demonstrated in an experimental model and in a controlled clinical trial.

## Results

### RBCs release a cardioprotective factor during hypoxia.

**Figure 1:**
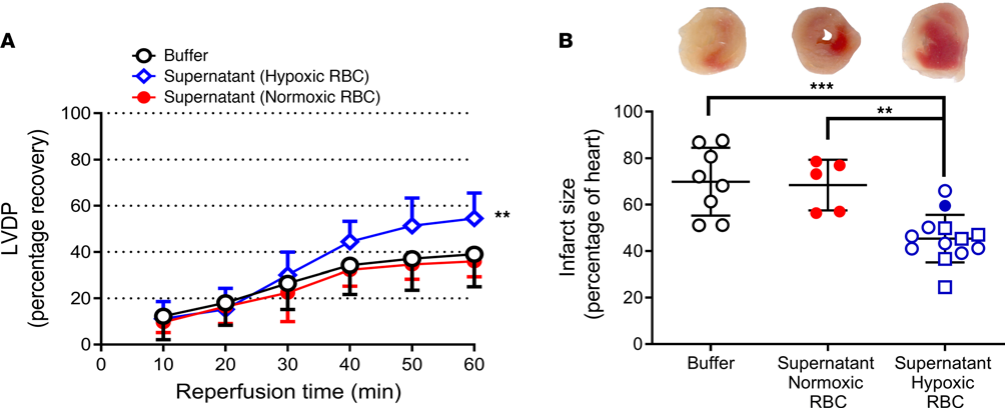
RBCs release a cardioprotective factor during hypoxia. (**A**) Percentage recovery of LVDP during reperfusion and (**B**) infarct size following administration of KH buffer (*n* = 8), supernatant from normoxic RBCs (*n* = 5), and hypoxic RBCs (*n* = 13). The recovery of LVDP is expressed as percentage of the preischemic level. The infarct size is presented as percentage of the whole heart. Data are presented as mean ± SD. The data points in the Buffer group are the same as those in the Figure 3 Buffer group. Data points in the Supernatant Hypoxic RBC group in panel **B** shown as open circles are also presented in Figure 2D and Figure 4D, and the filled circle is also shown in Figure 2D, whereas data points shown as open squares are only presented in this figure. The same overlapping data points in the Supernatant (Hypoxic RBC) group in panel A shown are also included in Figure 2C and Figure 4C. **P < 0.01 and ***P < 0.001 denote significant differences to buffer using 2-way ANOVA followed by Tukey’s multiple comparison test in **A** and 1-way ANOVA followed by Tukey’s multiple comparison test in **B**.

First, we investigated whether RBCs exposed to hypoxia release a cardioprotective mediator by administration of RBC supernatant to an isolated heart subjected to global IR. To this end, the supernatant collected from mouse RBCs exposed to normoxia or hypoxia was given to mouse hearts at the onset of ischemia. The supernatant from RBCs exposed to hypoxia significantly improved postischemic cardiac recovery of left ventricular developed pressure (LVDP) (Figure 1A) and reduced infarct size (Figure 1B) compared with supernatant from RBCs exposed to normoxia or Krebs-Henseleit (KH) buffer. Reoxygenation of the supernatant collected from hypoxic RBCs failed to attenuate the protective effect, and low oxygen KH buffer (without RBCs) collected following a 1-hour incubation in hypoxic conditions did not exert any cardioprotective effect (Supplemental Figure 1, A and B; supplemental material available online with this article; https://doi.org/10.1172/JCI167693DS1). These data suggest that the protection induced by exposing the RBC to hypoxia is independent of the supernatant oxygen level per se but rather mediated by a factor released from the RBCs. The protective effect of the supernatant from hypoxic RBCs was not affected by passing it through a 100 kDa filter (Supplemental Figure 1C), suggesting that extracellular vesicles were not involved in the protection. Nor was it affected by exposure to UV light (Supplemental Figure 2), suggesting that light sensitive S-nitrosothiols were not involved.

### The cardioprotective effect of hypoxia is mediated by RBC sGC and export of cGMP.

**Figure 2:**
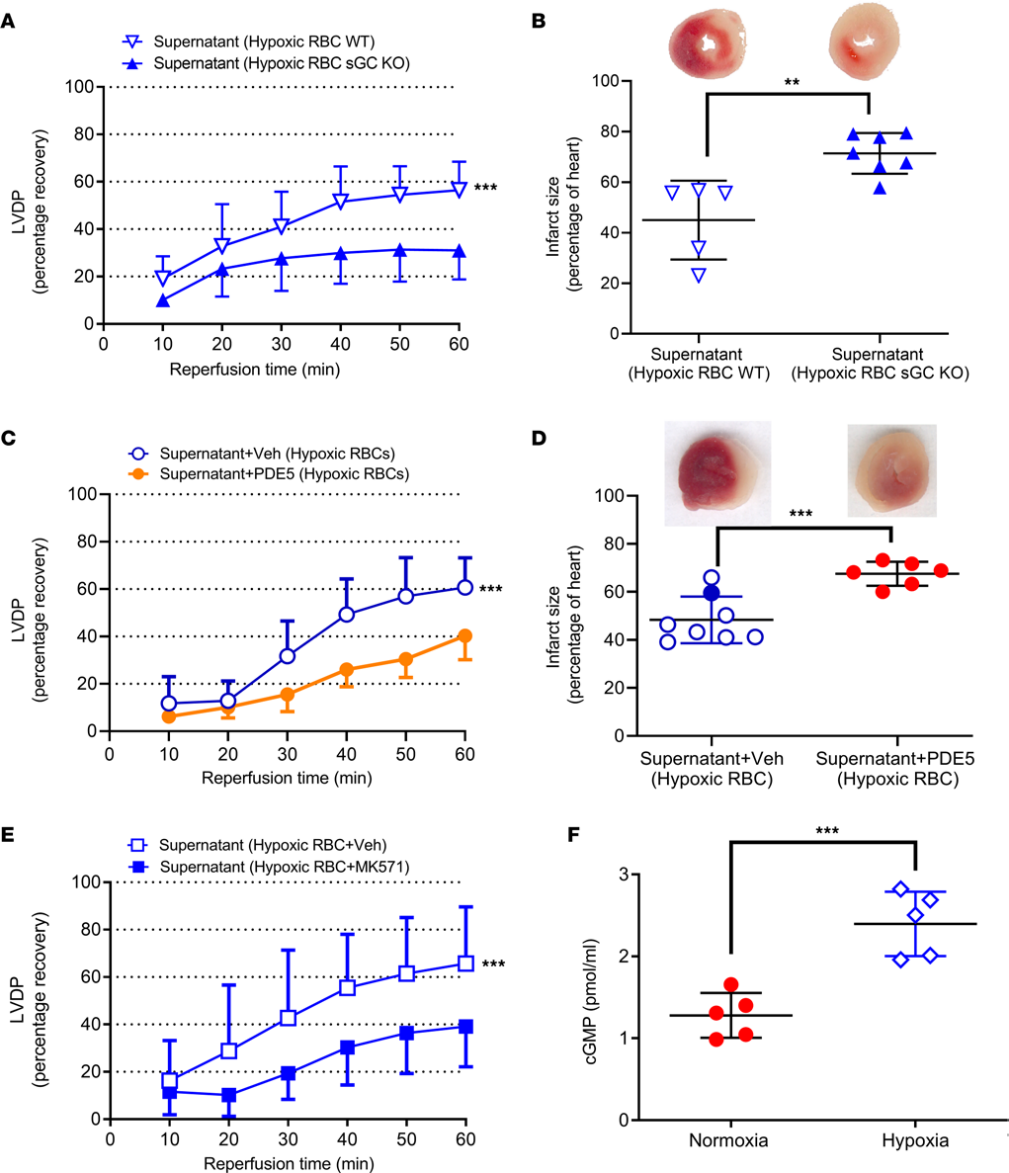
Hypoxia-induced release of cardioprotective cGMP from RBCs. (**A**) Percentage recovery of LVDP and (**B**) infarct size following administration of supernatant from hypoxic RBCs from WT mice (*n* = 5) or sGC KO mice (*n* = 7). (**C**) Recovery of LVDP and (**D**) infarct size following administration of supernatant from hypoxic RBCs incubated with vehicle(veh; *n* = 8) or recombinant PDE5 (*n* = 6). (**E**) Recovery of LVDP following administration of supernatant from hypoxic RBCs incubated with vehicle (*n* = 11) or the cGMP transport inhibitor MK571 (*n* = 5). (**F**) cGMP in supernatant of RBCs exposed to normoxia and 1 hour of hypoxia. Data are presented as mean ± SD. Data points in the **D** Supernatant Hypoxic RBC+Veh group are also shown as open and filled circles in 1B and open circles in the Figure 4D Supernatant Hypoxic RBC group. The same overlapping data points in the Supernatant Hypoxic RBC group in **C** are also included in Figures 1A and 4C in the Supernatant Hypoxic RBC group. **P < 0.01 and ***P < 0.001 denote significant differences between groups using 2-way ANOVA followed by Tukey’s multiple comparison test in **A**, **C** and **E**, Mann-Whitney test in **B**, and an unpaired *t* test in **D** and **F**.

Having determined that a cardioprotective factor was released from RBCs during hypoxia, we next sought to determine the nature of this compound. The focus was directed toward sGC-cGMP as this pathway has been shown to be present in RBCs. To determine the involvement of sGC, we exposed RBCs collected from mice lacking the α1-subunit of sGC (GC KO mice) to hypoxia and the supernatant was administered to hearts from WT mice. The supernatant from these RBCs exposed to hypoxia failed to protect the hearts against IR injury, strongly suggesting that RBC sGC played a key role in cardioprotection (Figure 2, A and B).

**Figure 3:**
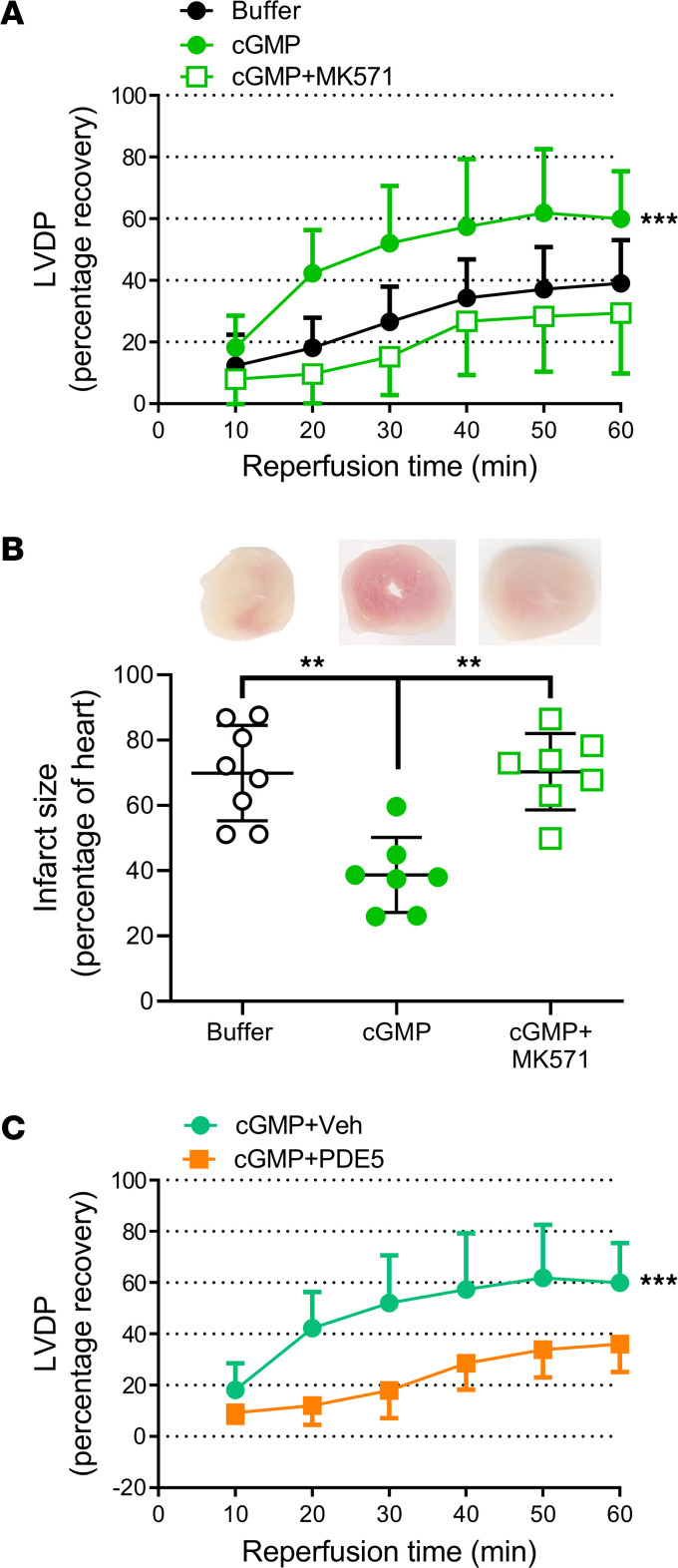
cGMP induces cardioprotection. (**A**) Percentage recovery of LVDP and (**B**) infarct size after administration of buffer (*n* = 8), cGMP (0.1 mM, *n* = 7) in buffer, or cGMP following administration of MK571 (*n* = 7). (**C**) Recovery of LVDP after administration of cGMP (0.1 mM, *n* = 7) in buffer or following administration of recombinant PDE5 (*n* = 6). Data are presented as mean ± SD. The data points in the Buffer group are the same as those in the Figure 1 Buffer group. **P < 0.01 and ***P < 0.001 denote significant differences between groups or compared with vehicle in **A** using 2-way ANOVA followed by followed by Tukey’s multiple comparison test in **A** and **C** and 1-way ANOVA followed by Tukey’s multiple comparison test in **B**.

Having confirmed that RBC sGC was responsible for the observed effects, we next performed experiments to determine if cGMP, the product of sGC, is exported from the RBC. To determine the involvement of cGMP in the cardioprotective effect of the supernatant from hypoxic RBCs, the cGMP hydrolyzing enzyme phosphodiesterase 5 (PDE5) was administered to the supernatant before administration of the supernatant to the isolated heart. The cardioprotective effect in the presence of PDE5 was compared to a control group given supernatant from RBCs exposed to hypoxia without addition of PDE5 and performed during the same time period. Importantly, administration of PDE5 abolished the cardioprotective effect of the supernatant from hypoxic RBCs (Figure 2, C and D), clearly supporting a role of cGMP. In additional experiments, RBCs from WT mice were incubated with MK571, an inhibitor of multidrug resistance proteins (MRP) type 4 and 5 which are responsible for membrane transport of cGMP. Coincubation with MK571 also blocked the cardioprotective effect of the supernatant from hypoxic RBCs (Figure 2E). Finally, detection of cGMP in the supernatant collected from RBCs revealed that cGMP levels were elevated in supernatant collected from RBCs exposed to hypoxia (Figure 2F). The cardioprotective effects of the supernatant were mimicked by administration of exogenous cGMP to the hearts at the onset of ischemia (Figure 3, A and B and Supplemental Figure 3), and this effect was, similar to that of the supernatant, abolished by PDE5 (Figure 3C) and by MK571 (Figure 3, A and B). Collectively, these observations suggest that RBCs exported cardioprotective cGMP.

### Dietary nitrate augments RBC-induced hypoxic cardioprotection.

**Figure 4:**
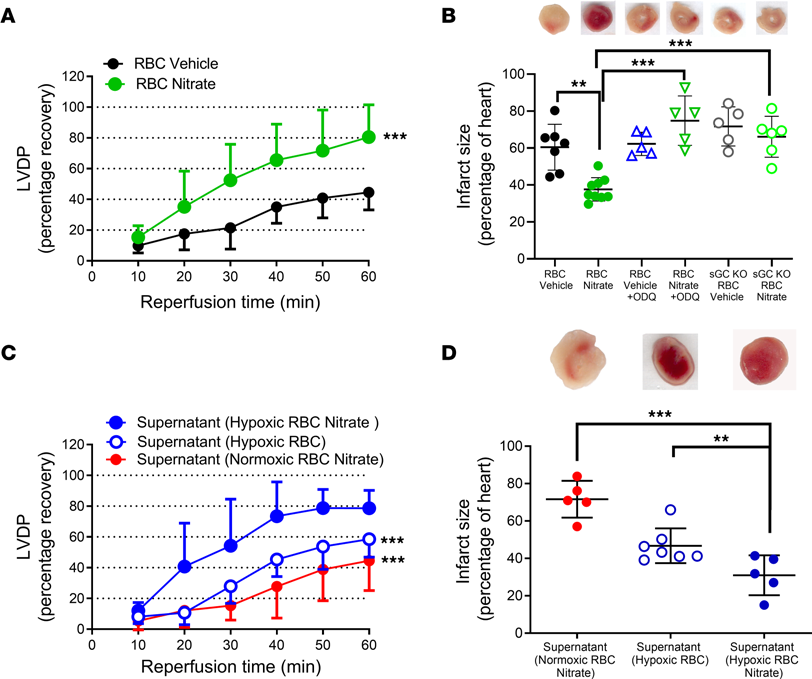
RBCs release a cardioprotective factor dependent on sGC following nitrate administration and hypoxia. Percentage recovery of LVDP during reperfusion (**A** and **C**) and infarct size (**B** and **D**) following administration of RBCs or RBC supernatant from mice given vehicle or oral nitrate (NO_3_^–^) for 4 weeks. (**A**) LVPD following administration of RBCs from vehicle (*n* = 7) and nitrate-treated (*n* = 9) mice. (**B**) Infarct size following administration of RBCs from vehicle-treated and nitrate-treated WT mice under control conditions (*n* = 7 and 9, respectively) or following incubation with the sGC inhibitor ODQ (*n* = 5), and RBCs from vehicle- and nitrate-treated sGC KO mice (*n* = 5 and 6, respectively). **C** and **D** depict effects of supernatant from RBCs from nitrate-treated mice and exposed to normoxia (*n* = 5) or hypoxia (*n* = 5), and supernatant from RBCs from vehicle-treated mice and exposed to hypoxia (*n* = 7). The recovery of LVDP is expressed as percentage of the preischemic level. The infarct size is presented as percentage of the whole heart. Data are presented as mean ± SD. Data points in the Supernatant (Hypoxic RBC) group in **D** are also shown as open circles in Figures 1B and 2D Supernatant Hypoxic RBC groups. The same overlapping data points in the Supernatant Hypoxic RBC group in **C** are also included in the Figures 1A and 2C Supernatant Hypoxic RBC groups. **P < 0.01 and ***P < 0.001 denote significant differences to the vehicle group by 2-way ANOVA in **A**, to supernatant from RBCs from nitrate-treated mice and exposed to hypoxia by 2-way ANOVA followed by Tukey’s multiple comparison test in **C**, and 1-way ANOVA followed by Tukey’s multiple comparison test in **B** and **D**.

Inorganic nitrate can be reduced to nitrite and deoxygenated hemoglobin can further reduce nitrite to NO (3, 10). Therefore, to further boost the RBC NO-sGC signaling pathway, mice were treated with inorganic nitrate or vehicle in the drinking water for 4 weeks, after which the RBCs were collected and given to isolated hearts subjected to IR. Hearts receiving RBCs from nitrate-treated mice had significantly better postischemic recovery of LVDP and smaller infarct size compared with hearts given RBCs from vehicle-treated mice (Figure 4, A and B). As in the experiments described above, we next tested whether dietary nitrate would augment hypoxia-induced export of the cardioprotective factor from RBCs. For these comparisons, the cardioprotective effect was compared both to a group exposed to supernatant from normoxic RBCs collected from nitrate-fed mice and to supernatant of hypoxic RBCs from control mice (normal drinking water) investigated during the same time period. Indeed, the supernatant from these RBCs provided strong protection against IR injury (Figure 4, C and D). In fact, the cardioprotective effect of nitrate and hypoxia was greater than that of hypoxia alone (Figure 4, C and D). By contrast, the supernatant from normoxic RBCs collected from nitrate-treated mice did not protect the heart against IR injury (compare Figure 1 and Figure 4, C and D). Collectively, these results demonstrate that the cardioprotection induced by hypoxic RBCs was augmented by dietary inorganic nitrate.

**Figure 5:**
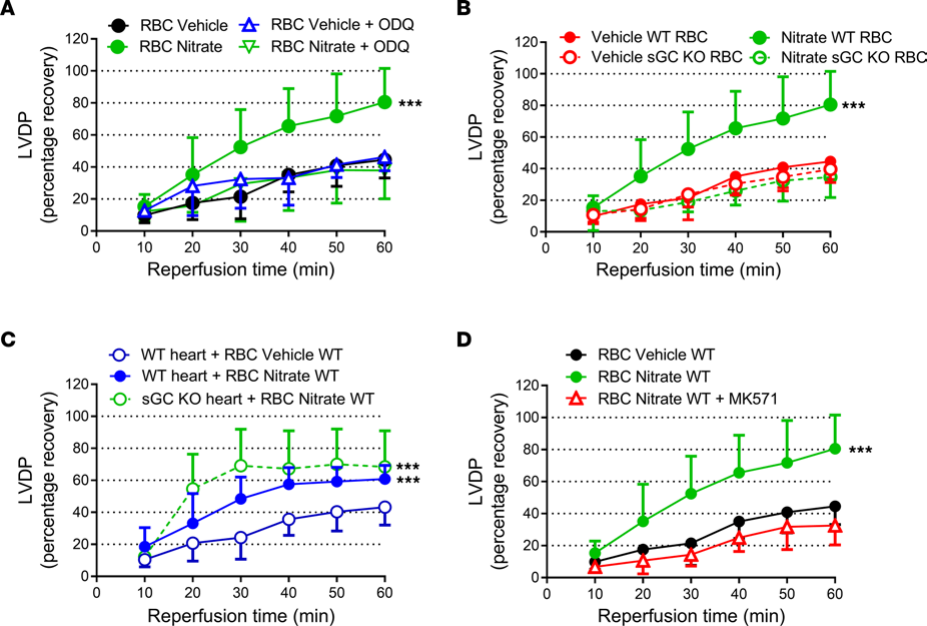
The nitrate-mediated protective effect of RBCs is dependent on sGC and transport by MRP. Percentage recovery of LVDP during reperfusion (**A**) following administration of RBCs from vehicle-treated and nitrate-treated mice under control conditions (*n* = 7 and 9, respectively) and following incubation with the sGC inhibitor ODQ (*n* = 5) or (**B**) following administration of RBCs from vehicle-treated and nitrate-treated control WT mice (*n* = 7 and 9, respectively) and from sGC KO mice (*n* = 15 and *n* = 8, respectively). (**C**) Recovery of LVDP following administration of RBCs from vehicle-treated and nitrate-treated WT mice to hearts from WT mice (*n* = 9 and 6, respectively), and nitrate-treated WT mice to hearts from sGC-KO mice (*n* = 5) and (**D**) vehicle-treated and nitrate-treated mice (*n* = 7 and 9, respectively) following incubation with the MRP-inhibitor MK571 (*n* = 4). The recovery of LVDP is expressed as percentage of the preischemic level. Data are presented as mean ± SD. ***P < 0.001 denotes significant differences to the vehicle group by 2-way ANOVA followed by Tukey’s multiple comparison test.

To identify the mechanism by which nitrate induces protection via RBCs, we again focused on the sGC-cGMP pathway. In these experiments, control RBCs collected from mice given normal drinking water (vehicle) or nitrate for 4 weeks were compared both with RBCs incubated with the sGC inhibitor ODQ before being administered to the isolated hearts and with RBCs from sGC-KO mice treated with nitrate. The control experiments and those with incubation with ODQ were matched in time, whereas the experiments using RBCs from sGC-KO mice were run at a separate occasion due to limitation in availability of the KO mice. Preincubation of the RBCs with ODQ blocked the protective effect induced by RBCs from nitrate-treated mice (Figure 4B and Figure 5A). In contrast, administration of ODQ selectively into the heart by a 10 minute infusion before the administration of the RBCs failed to affect the protection induced by RBCs from nitrate-treated mice (Supplemental Figure 4), suggesting that cardiac sGC signaling was of minor importance for the observed effects. Next, we investigated the cardioprotective effect of RBCs from sGC-KO mice. RBCs harvested from nitrate-treated sGC-KO mice failed to protect normal WT hearts from IR injury (Figure 4B and 5B). By contrast, RBCs from nitrate-treated WT mice were able to protect hearts from sGC-KO mice against IR injury (Figure 5C), again demonstrating that cardiac sGC signaling is not needed for the observed protection. Finally, preincubation of RBCs with the cGMP transport inhibitor MK571 again abolished the cardioprotection induced by RBCs from nitrate-treated mice (Figure 5D). In aggregate, these data demonstrate that dietary intervention with nitrate affords protection from IR injury via activation of an sGC signaling pathway in the RBC. Surprisingly, this signaling does not require an intrinsic sGC pathway in the heart itself.

### Cardiac protection of RBCs is PKG-dependent.

**Figure 6:**
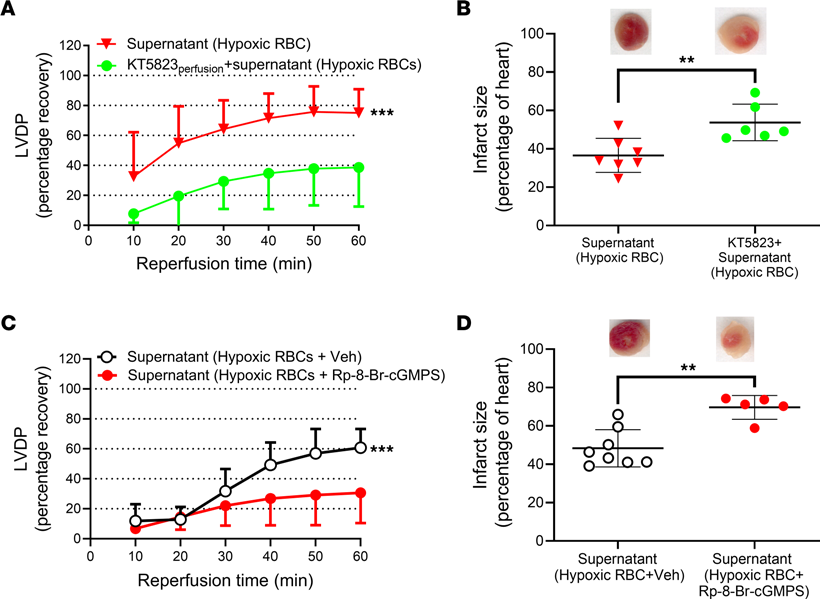
RBCs mediate cardioprotection via activation of cardiac PKG. (**A**) Percentage recovery of LVDP during reperfusion and (**B**) infarct size following administration of supernatant collected from hypoxic RBCs and administration of supernatant following perfusion of the heart with the PKG inhibitor KT5823 (1 μM; KT5823perfusion; *n* = 6). (**C**) Recovery of LVDP and (**D**) infarct size following administration of supernatant collected from hypoxic RBCs and vehicle (*n* = 8) or with the PKG inhibitor Rp-8-Br-cGMPS (*n* = 5). Data are presented as mean ± SD. **P < 0.01 and *** *P* < 0.001 denote significant differences between groups or to the vehicle group by 2-way ANOVA followed by Tukey’s multiple comparison test in **A** and **C** and unpaired *t* test in **B** and **D**.

**Figure 7:**
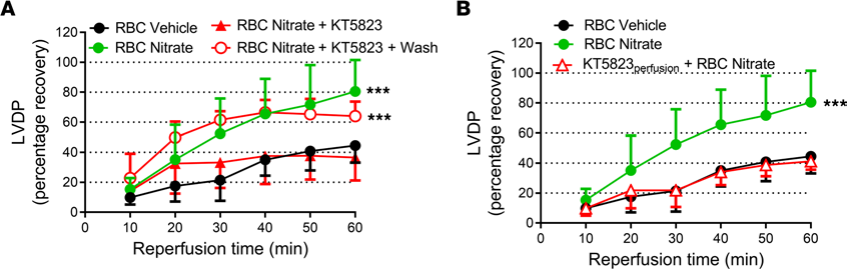
Inhibition of cardiac protein kinase G abolishes cardioprotection induced by RBCs from nitrate-treated mice. Percentage recovery of LVDP during reperfusion following administration of RBCs from (**A**) vehicle-treated mice (RBC Vehicle, *n* = 7), nitrate-treated mice (RBC Nitrate, n = 9), nitrate-treated mice following incubation with the PKG inhibitor KT5823 (10 μM; *n* = 4), and nitrate-treated mice following incubation KT5823 and washing of the RBCs to remove extracellular KT5823 (+Wash, *n* = 5) and (**B**) vehicle-treated mice (*n* = 7), nitrate-treated mice (*n* = 9), and nitrate-treated mice following perfusion of the hearts with KT5823 before administration of the RBCs (KT5823perfusion, *n* = 5). Data are presented as mean ± SD. ***P < 0.001 denotes significant differences between groups or to the vehicle group by 2-way ANOVA followed by Tukey’s multiple comparison test.

**Figure 8:**
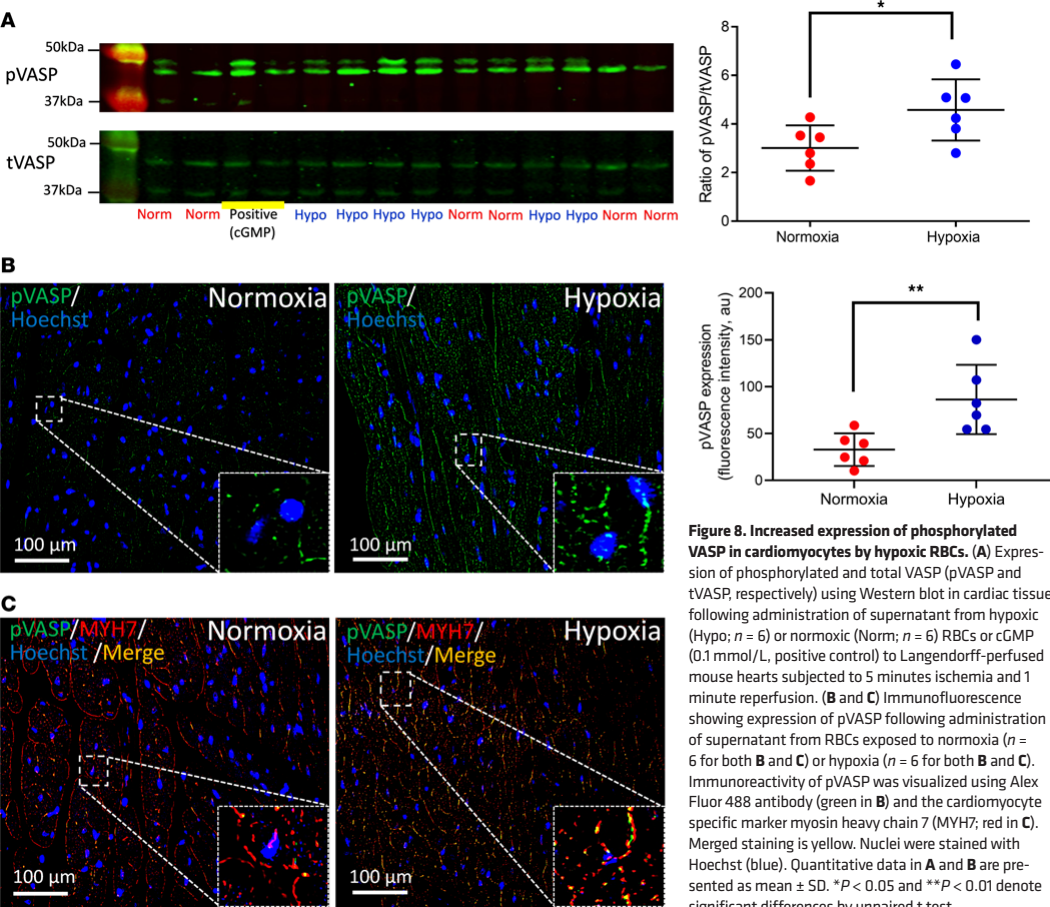
Increased expression of phosphorylated VASP in cardiomyocytes by hypoxic RBCs. (**A**) Expression of phosphorylated and total VASP (pVASP and tVASP, respectively) using Western blot in cardiac tissue following administration of supernatant from hypoxic (Hypo; *n* = 6) or normoxic (Norm; *n* = 6) RBCs or cGMP (0.1 mmol/L, positive control) to Langendorff-perfused mouse hearts subjected to 5 minutes ischemia and 1 minute reperfusion. (**B** and **C**) Immunofluorescence showing expression of pVASP following administration of supernatant from RBCs exposed to normoxia (*n* = 6 for both **B** and **C**) or hypoxia (*n* = 6 for both **B** and **C**). Immunoreactivity of pVASP was visualized using Alex Fluor 488 antibody (green in **B**) and the cardiomyocyte specific marker myosin heavy chain 7 (MYH7; red in **C**). Merged staining is yellow. Nuclei were stained with Hoechst (blue). Quantitative data in **A** and **B** are presented as mean ± SD. *P < 0.05 and **P < 0.01 denote significant differences by unpaired t test.

After having established the signaling pathway in RBCs under hypoxia and following nitrate administration, we investigated the downstream signaling in the heart focusing on protein PKG, the major target of cGMP. In these experiments we used 2 distinct inhibitors of PKG. In a first set of experiments, the inhibitor of cGMP-binding to PKG, Rp-8-bromoguanosine-3′,5′-cyclic monophosphorothioate (Rp-8-Br-cGMPS) (17, 18), or the PKG inhibitor KT5823 were added to the supernatant from RBCs following exposure to hypoxia. The cardioprotective effect of the supernatant from hypoxic RBCs was abolished by both KT5823 (Figure 6, A and B) and Rp-8-Br-cGMPS (Figure 6, C and D), suggesting cGMP-dependent activation of PKG by the mediator released from RBCs. The cardioprotective effect of RBCs from nitrate-treated mice was also blocked by coadministration of the PKG inhibitor KT5823 (Figure 7A). Conversely, when RBCs from nitrate-treated mice were preincubated with the PKG inhibitor followed by extensive RBC washing and removal of extracellular KT5823, the RBCs still induced cardioprotection (Figure 7A). Next, to distinguish between activation of PKG in the heart and in RBCs, cardiac PKG was blocked by perfusing the heart with KT5823 for 10 minutes before the administration of RBCs from nitrate-treated mice. In this situation, the RBCs from nitrate-treated mice failed to protect the heart (Figure 7B). In addition, administration of the supernatant from RBCs exposed to hypoxia resulted in increased PKG-dependent phosphorylation of cardiac vasodilator-specific phosphoprotein (VASP) compared with supernatant from normoxic RBCs (Figure 8A). Finally, detection of pVASP using immunofluorescence revealed that administration of supernatant from hypoxic RBCs induced a clear-cut increase in cardiac pVASP compared with supernatant from normoxic RBCs (Figure 8B). This increased expression of pVASP was colocalized with the cardiomyocyte marker myosin heavy chain 7 (Figure 8C) indicating presence in cardiomyocytes. The changes in pVASP induced by the supernatant were comparable to those induced by exogenous cGMP (Supplemental Figure 5). These data suggest that the cardioprotective effect of RBCs from nitrate-treated mice and hypoxic RBCs was mediated via cardiomyocyte PKG.

### Pharmacological sGC stimulation in RBCs also induces cardioprotection and cGMP release.

To verify that pharmacological activation of RBC sGC produce effects similar to those of hypoxia, RBCs from WT and sGC-KO mice were preincubated with the sGC stimulator BAY 41-2272 in combination with the NO donor DEA/NO and the PDE5 inhibitor sildenafil before administration to the isolated heart. Preincubation of RBCs from WT mice resulted in improved recovery of postischemic LVDP, and this effect was blocked by the sGC inhibitor ODQ and was absent when using RBCs from sGC-KO mice (Supplemental Figure 6). Further, incubation of RBCs from WT mice with the sGC stimulator vericiguat increased extracellular cGMP levels (Supplemental Figure 7). These results suggest that the effects of hypoxia on cardiac recovery and release of cGMP can be reproduced by pharmacological stimulation of sGC.

### RBCs from nitrate-treated humans induce cardioprotection.

**Figure 9:**
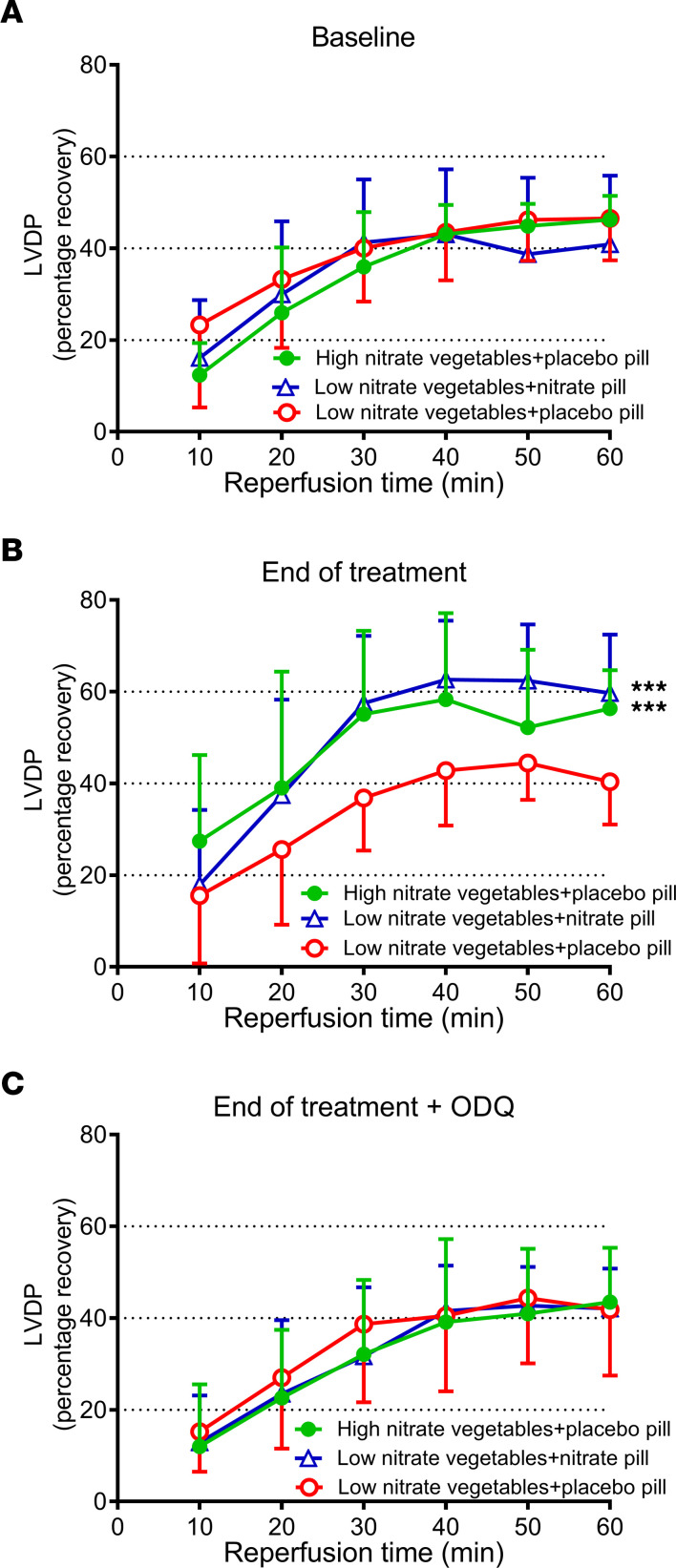
Dietary nitrate in humans enables RBCs to mediate cardioprotection. RBCs collected from 2 groups of subjects randomized to high nitrate intake in the form of nitrate pills or nitrate-rich vegetables and 1 group subjected to low dietary intake of nitrate were given to isolated rat hearts subjected to ischemia reperfusion. The RBCs were investigated (**A**) at baseline before randomization (*n* = 14–16 in each group), (**B**) at end of treatment (*n* = 14–16), and (**C**) at end of treatment following incubation of the RBCs with the sGC inhibitor ODQ (*n* = 11–13). Data show percentage recovery of LVDP during reperfusion and are presented as mean ± SD. ***P < 0.001 denotes significant differences to the low nitrate vegetables+placebo pill group by 2-way ANOVA followed by Tukey’s multiple comparison test.

To translate the beneficial effect of nitrate administration in mice to the clinical situation, RBCs were collected from 3 groups of subjects randomized to a 5-week dietary intervention: 2 groups with high nitrate in the form of a potassium nitrate pill or nitrate-rich vegetables and 1 group receiving a low dietary intake of nitrate (19). These RBCs were then given to isolated rat hearts subjected to IR. At baseline before randomization, the recovery of postischemic LVDP of hearts given RBCs from the 3 groups was comparable (Figure 9A). Importantly, at the end of the intervention period, RBCs from both high-nitrate intake groups significantly improved postischemic cardiac recovery compared with RBCs from the low nitrate intake group (Figure 9B). Interestingly, the magnitude in cardioprotection induced by RBCs was similar in the groups receiving nitrate in the form of vegetables and potassium nitrate pills. Further, the cardioprotective effect was abolished by preincubating the RBCs with the sGC inhibitor ODQ (Figure 9C), again suggesting that sGC in RBCs played a critical role in the cardioprotective effect.

## Discussion

Here, we show that RBCs under hypoxic conditions induce cardioprotection through a mechanism that is dependent on sGC in the RBC and associated with export of cGMP that, via a paracrine effect, is involved as a mediator of the cardioprotection through activation of PKG in the heart. This cardioprotective effect is boosted by a dietary intervention with inorganic nitrate. The strict involvement of RBC sGC in the cardioprotective effect was demonstrated using both pharmacological and gene-targeted approaches. Finally, we demonstrate that this cardioprotective signaling by RBCs also occurs in humans following intake of dietary nitrate in amounts readily achieved through a rich intake of green leafy vegetables.

It has been suggested that RBCs are capable of inducing hypoxic vasodilatation through the generation of a signal during deoxygenation of hemoglobin (1) and of protecting the heart during IR (13). A key finding of the present study is that RBCs exposed to hypoxia release a cardioprotective mediator that is transferable to the heart to induce protection against IR injury. These results suggest that RBCs under hypoxic conditions not only induce vasodilatation (1, 2) but also possess the important ability to mediate tissue protection during ischemia. This finding significantly advances our understanding regarding the role of the RBC during pathological situations. Using both pharmacological inhibition and KO mice, we provide several lines of evidence suggesting that the cardioprotective mediator released by RBCs under hypoxic conditions is cGMP derived from sGC-activation. An important finding is that the cardioprotective effect of the supernatant from hypoxic RBCs was abolished by the cGMP-hydrolyzing enzyme PDE5, which clearly suggests that cGMP is involved as a mediator of the cardioprotection. The protection was also blocked by MK571, an inhibitor of MRP types 4 and 5, as well as organic anion transporters (OAT), which are known to be responsible for transport of cGMP (20–22), suggesting that this protection involves active transport of cGMP. Accordingly, cGMP levels in supernatant collected from RBCs were significantly elevated following exposure of the RBCs to hypoxia. It is important to note that all of these findings using the supernatant of hypoxic RBCs, including cardioprotection and sensitivity to PDE5 and MK571, were fully reproduced by exogenous cGMP. The finding that the cardioprotective effect of exogenous cGMP, like that of the supernatant from hypoxic RBCs, was blocked by MK571 is in line with previous observations that this compound interferes not only with export of cGMP but also with transport of cGMP into cells (22, 23). The data also indicate that cGMP exerts cardioprotection via intercellular signaling in addition to its well-known intracellular signaling. The observation that cGMP may exert a paracrine effects is, to some extent, supported by the earlier finding that release of cGMP from mucosal epithelial cells acts on nociceptors to reduce nociception (24). Moreover, it was recently suggested that cGMP can be transported between cardiac fibroblasts and cardiomyocytes via gap junctions (25). Our results give further support to the increasing interest in cGMP-modulating drugs in cardiovascular disease (26) by demonstrating that cGMP is exported from RBCs and induces cardioprotection. Further, our data provide a possible explanation for how RBC-derived NO signaling can escape scavenging by hemoglobin to exert functional responses in another tissue/organ.

We next hypothesized that dietary supplementation with inorganic nitrate — a precursor of nitrite that is further metabolized in blood (3) and tissues (27) to NO and other bioactive nitrogen oxides — results in enhanced cardioprotective properties of RBCs. It has been shown that inorganic nitrate, after initial reduction to nitrite, results in increased NO formation leading to beneficial cardiovascular effects, including blood pressure reduction and protection against myocardial IR injury (27–29). This nitrate-nitrite-NO pathway is emerging as an important complement to the classical NOS-dependent production, especially in circumstances when NOS activity is compromised, as in hypoxic/ischemic conditions (28, 30). RBCs can also transport nitrite and nitrate and are, therefore, considered a major source of stored nitrite in the circulation (31). In line with this, we observed a clear cardioprotective effect of RBCs collected from mice that had been given oral nitrate. Further, the cardioprotective effect of inorganic nitrate was markedly enhanced by exposing the RBCs to hypoxia, supporting the notion that increasing the NO signaling pathway can further augment the protective signaling in RBCs and the export of the protective mediator. Several lines of evidence presented in this study suggest that this effect was mediated via activation of RBC sGC, export of a cardioprotective mediator,and activation of PKG in cardiomyocytes.

An interesting issue is how hypoxia leads to sGC activation in the RBC with downstream cGMP signaling resulting in cardioprotection. RBCs are known to be a major source of nitrite (31), and nitrite reductase activity of hemoglobin is modulated by deoxygenation (9). Thus, it is possible that hypoxia leads to increased RBC NO formation from nitrate via formation of nitrite that is further reduced to NO with subsequent activation of sGC. This would also explain the augmentation of the cardioprotection following oral administration of nitrate in combination with hypoxia. The importance of hypoxia would also explain why administration of normoxic RBCs but not the supernatant from normoxic RBCs protected the heart during ischemia. It may be speculated that the model of cardiac ischemia represents a pronounced hypoxic environment for the RBCs, leading to formation of NO bioactivity from nitrite with downstream activation of sGC in the RBC, and subsequent extracellular transport of a signal leading to activation of PKG in the cardiac tissue, as shown by the increased cardiac phosphorylation of VASP (32). Alternatively, there may be other forms of “stored NO bioactivity” in the RBC whose release is triggered by hypoxia. It was recently shown that heme-free inactive sGC may be activated by NO-ferroheme (33). Therefore, an alternative explanation may be that nitrate supplementation increases formation of NO-ferroheme and leads to activation of a heme-free sGC.

A major strength of the present study is that we were able to translate these findings to the clinical setting with validation of the results in RBCs from patients with hypertension. RBCs collected from patients on a high nitrate diet afforded marked sGC-dependent cardioprotection compared with RBCs from the group receiving a low-nitrate diet. The cardioprotection was of comparable magnitude irrespective of whether nitrate was given in the form of nitrate pills or nitrate-rich vegetables, strongly supporting that the effect was mediated by nitrate per se. The observation that dietary nitrate induces RBC-mediated protection against cardiac IR injury in this patient group is of interest considering the importance of hypertension as a risk factor for myocardial infarction. It should be emphasized that the amount of nitrate was not more than what can readily be consumed with a normal diet. To study if this cardioprotective effect of nitrate is seen also in vivo in humans will obviously require a prospective clinical trial. Nevertheless, it is interesting that green leafy vegetables (the major dietary nitrate source) constitute a central part of diets associated with reduction in cardiovascular events (34).

This study, along with our earlier studies (13, 14), demonstrate that neither eNOS nor sGC needs to be present in the heart itself for cardioprotection. Instead, the RBCs provide both the NO signaling needed to activate sGC and the second messenger, cGMP, to activate cardiac PKG. So why then is NO not generated in the heart itself during hypoxia to stimulate local generation of cGMP? One might speculate about the reasons for this. First, eNOS cannot generate any NO when oxygen levels in the heart fall below a critical level, while generation of NO bioactivity in the RBC from, for example, nitrite is instead accelerated under these conditions. Second, it may be that too much NO in the cardiomyocytes would be detrimental given the potent inhibitory effects of NO on mitochondrial respiration (35). Last, by avoiding too much NO in the cardiac muscle during reperfusion, potential oxidant damage may be dampened due to less formation of reactive nitrogen species, including peroxynitrite.

This study has certain limitations that need to be considered. First, the cardioprotective effects of the RBCs are demonstrated using RBCs ex vivo in an isolated heart model. It remains to be demonstrated to what degree NO bioactivity derived from hypoxic RBCs may mediate cardioprotection in vivo. Earlier studies have shown that oral administration of nitrite and nitrate protects the heart from IR injury (29, 36). However, to distinguish the cardioprotective effect of NO bioactivity derived from RBCs exposed to hypoxia from signaling derived from other cells under in vivo conditions is, unfortunately, very challenging. A recent study demonstrated that RBCs collected from patients during acute myocardial infarction induced cardioprotection (37), supporting that the phenomenon revealed here may be of relevance in the clinical setting of myocardial infarction. The strength with the ex vivo model is that it permits determination of the specific actions of RBCs on cardiac function. However, any extrapolation of the present findings to the in vivo setting should be made with caution. Second, the levels of extracellular cGMP detected following hypoxia are lower than those of exogenous cGMP needed to induce cardioprotection, and it may be argued that the levels of endogenous cGMP are too low to induce cardioprotection. This raises the question of whether additional mediators related or unrelated to cGMP also are in play. Nevertheless, the involvement of endogenous cGMP is supported by the complete dependence of sGC for the response, the complete inhibition of cardioprotective activity by addition of recombinant PDE5, the involvement of a cGMP transport system, and the activation of cGMP-dependent PKG. However, it is still possible that additional mediators, including biologically active derivatives of cGMP such as nitro-cGMP (38) or SH-cGMP (39), may be formed under these experimental conditions and contribute to the observed effects. Finally, all studies were conducted only on male mice, and the absence of a female cohort is a limitation of the study.

In conclusion, the present results reveal a fundamental endogenous protective mechanism mediated by RBCs when exposed to hypoxic conditions. To the best of our knowledge, this is the first demonstration of intercellular or paracrine signaling by cGMP exported from hypoxic RBCs. The abundance and ubiquitous presence of RBCs make them highly suitable for the purpose of tissue protection, suggesting that this may represent a physiological defense mechanism designed to minimize IR injury. This finding may provide an explanation to the recently observed cardioprotective effect of RBCs collected from patients with acute myocardial infarction (37). It will be of interest to study whether RBC-mediated cGMP export is involved also in other physiological effects modulated by the nitrate-NO-sGC-cGMP pathway, including vasodilation (9), control of blood pressure (40),and metabolic function (41). Moreover, the therapeutic and preventive effects of boosting RBC-mediated tissue protection with simple dietary interventions are potentially important and should be evaluated further.

## Methods

### Animals.

Wistar rats were purchased from Charles River and used for experiments at 12 to 14 weeks of age. C57Bl/6J mice were purchased from Janvier and used for experiments at 8 to 15 weeks of age. Mice lacking the α1-subunit of sGC — and thus α1β1-sGC (α1-GC KO mice) — were generated and genotyped as described previously (42). Maintenance of the colony was achieved by backcrossing heterozygous (a1+/—GC) mice to WT mice (C57Bl/6Rj). The resulting F1 compound heterozygotes were then intercrossed to generate F2 mice (KO and the respective WT strain in parallel). The KO and WT mice analyzed in the present study were F2 generation, 4 to 8 months old, and males and were backcrossed to C57Bl/6Rj background for 18 to 20 times (N18–N20 generation). The mice were held in a conventional mouse facility in a 12 hour light-dark cycle at 22°C, 50%–60% humidity, and had access to food and water ad libitum.

### Heart isolation and perfusion.

Isolated hearts from rats (for human RBCs) or mice (for mouse RBCs) were perfused in a Langendorff system as described previously in detail (13, 43). Briefly, the animals were anesthetized (i.p.) with pentobarbital (50 mg/kg). Heparin (250 IU) was injected i.p. in mice and i.v. in rats. The hearts were excised and the ascending aorta was cannulated and perfused with oxygenated (5% CO_2_, 95% O_2_) modified KH buffer (in mM: NaCl 118.5, NaHCO_3_ 25.0, KCl 4.7, KH_2_PO_4_ 1.2, MgSO_4_ 1.2, glucose 11.1 and CaCl_2_ 2.4 [all from Sigma-Aldrich]) at constant pressure (70 mmHg and 90 mmHg for mouse and rat hearts, respectively) at 37°C. A balloon-tipped catheter was inserted into the left ventricle and connected to a pressure transducer for the recording of LVDP. The balloon was given a baseline of left ventricular end-diastolic pressure of 4–10 mmHg. The following inclusion criteria were used: left ventricular systolic pressure (LVSP) ≥60 mmHg and heart rate (HR) ≥280 bpm for mouse hearts and LVSP ≥80 mmHg and HR ≥250 bpm for rat hearts.

### RBC isolation.

Heparinized blood from mice and humans was centrifuged at 1,000*g* and 4°C for 10 minutes. Plasma and buffy coat were removed. The concentrated RBCs were washed 3 times by using KH buffer and centrifuged at 1,000*g* and 4°C for 5 minutes. The RBCs were diluted using KH buffer to hematocrit (Hct) about 40%.

### Supernatant of RBCs.

The RBCs suspension was exposed to hypoxia (1% O_2_ and 5% CO_2_) or normoxia (21% O_2_ and 5% CO_2_) at 37°C for 1 hour. The supernatant was collected following centrifugation at 1,000*g* and 4°C for 5 minutes.

### Nitrate treatment in mice.

C57Bl/6J mice at age of 8 weeks were housed under standard environmental conditions (room temperature at 22^o^C with a 12 hour light-dark cycle) and fed with a standard diet. All mice had free access to either nitrate-containing water (10 mM nitrate) or normal water (vehicle) for 4 weeks before taken into experiments.

### Experimental protocol.

After the start of perfusion, all hearts were allowed to stabilize for at least 30 minutes and baseline LVDP was registered. The duration of global ischemia, induced by clamping the inflow tubing, was 40 minutes for mouse hearts and 25 minutes for rat hearts (13). Reperfusion, initiated by releasing the clamp, was maintained for 60 minutes. At the start of ischemia, RBCs suspension (Hct approximately 40%) or supernatant were injected in volumes of 3 mL to rat hearts and 0.4 mL to mouse hearts into the coronary circulation via a sidearm of the perfusion system. In separate experiments, RBCs were preincubated with the following inhibitors (Sigma-Aldrich) of the sGC-PKG signaling pathway at 37°C for 25 minutes before being administered to the hearts: the sGC inhibitor 1H- [1,2,4] oxadiazolo [4,3,-a] quinoxalin-1-1 (ODQ; 5 μM), the cGMP transporter inhibitor 5-(3-(2-(7-Chloroquinolin-2-yl)ethenyl)phenyl)-8-dimethylcarbamyl-4,6-dithiaoctanoic acid (MK571; 10 μM), and the PKG inhibitor (9S,10R,12R)-2,3,9,10,11,12-Hexahydro-10-methoxy-2,9-dimethyl-1-oxo-9,12-epoxy-1H-diindolo[1,2,3-fg:3’,2’,1’-kl]pyrrolo[3,4-i][1,6]benzodiazocine-10-carboxylic acid (KT5823; 10 μM). Alternatively, the hearts were perfused for 10 minutes with the same inhibitors before RBCs administration. In additional experiments, isolated mouse hearts were pretreated with MK571 (10 μM), KT5823 (1 μM), or the PKG inhibitor (cGMP mimic) Rp-8-Br-cGMPS (10 μM; Sigma-Aldrich) via a side tubing of the perfusion system 10 minutes before administration of the supernatant collected from RBCs. Recombinant PDE5 (0.7 μM; Enzo) was added to the supernatant collected from RBCs 20 minutes before being administered to the isolated heart. Cyclic GMP (1 μM–1 mM; Sigma-Aldrich) was given to the heart in KH buffer only (without RBCs) or following pretreatment of the hearts with MK571 or PDE5 as described above. The concentrations used above were determined by pilot studies and according to a previous study (15). Any preparation that showed sign of hemolysis was excluded.

Additional experiments were performed using pharmacological stimulation of sGC in RBC from WT and sGC-KO mice. In these experiments, the RBCs were incubated with the sGC stimulator BAY 41-2272 (10 μM; Sigma-Aldrich), the PDE5 inhibitor sildenafil (100 μM; Sigma-Aldrich) and the NO donor DEA/NO (200 μM; Sigma-Aldrich) for 20 minutes before being administered to the isolated heart. Isolated RBCs from WT mice were also incubated with the sGC stimulator vericiguat (10 μM Bayer AG) for 1 hour. After centrifugation (1,000*g* and 4°C for 5 minutes), the supernatant was collected for determination of cGMP as described in detail below.

### Clinical study protocol.

Patients were recruited as a substudy of a double-blinded clinical trial investigating the effect of dietary nitrate on blood pressure in patients with mild hypertension (clinicaltrials.gov NCT02916615). The clinical trial was described in detail previously (19). Following a run-in period of 2 weeks with a low nitrate diet, the subjects were randomized into 1 of 3 groups: Group 1: Low-nitrate vegetables (tomato, sweet pepper, 150 g/day) + placebo pills (potassium chloride, twice daily) (*n* = 16); Group 2: low nitrate vegetables + nitrate pills (150 mg nitrate in the form of potassium nitrate, twice daily) (*n* = 16); or Group 3: leafy green vegetables (amount adjusted to contain 150 mg nitrate, twice daily) + placebo pills (*n* = 14). Compliance and amounts of nitrate ingested were confirmed by measurements of nitrate in the vegetables given and by nitrate measurements in urine collected over 24 hours (19). The subjects receiving low nitrate vegetables were double blinded and the subjects receiving leafy green vegetables were single blinded. The intervention was given for 5 weeks. The subjects were instructed to avoid all other vegetables in their diet during the entire study period. Blood samples were collected at baseline and at the end of treatment and RBCs were isolated for administration to rat hears subjected to IR as described above. The RBC suspension was incubated at 37°C with the sGC inhibitor ODQ or vehicle for 20 minutes and then given to the isolated rat hearts at the onset of ischemia.

### Determination of heart infarct size.

At the end of reperfusion, hearts were frozen at –20°C and sectioned into 1 mm–thick slices from the apex to the base, stained with triphenyltetrazolium chloride for 15 minutes, and fixed in 1% formaldehyde for 18 hours. Necrotic negatively stained myocardium was measured using Adobe Photoshop Elements 2019 Edition by an investigator blinded to group allocation (43).

### Expression of cardiac vasodilator specific phosphoprotein.

In separate experiments, isolated and perfused mouse hearts were subjected to 5 minutes of ischemia following 30 minutes of stabilization. The supernatant from hypoxic or normoxic mouse RBCs was given to the isolated heart at the start of ischemia. After 1 minute reperfusion, the hearts were harvested using liquid nitrogen and stored at –80°C. Protein was extracted from the heart by using radioimmunoprecipitation assay buffer (RIPA buffer, VWR International) containing protease inhibitors (Roche Diagnostics). The protocol of Western blotting was described previously (13). Briefly, proteins were separated by 12% SDS/PAGE, transferred to nitrocellulose membrane, and blocked in 5% nonfat milk for 1 hour at room temperature. Membranes were incubated overnight at 4°C with rabbit anti-pVASP (phosphorylation at Ser239, 1:1,000; catalog 3114, Cell Signaling Technology) following stripping of the membrane with a rabbit anti-VASP (1:1,000; catalog 3112, Cell Signaling Technology). Membranes were washed with TBS with 0.1% Tween 20 and incubated with a secondary antibody IRDye 107800CW goat anti-rabbit IgG (1:22,000; catalog 926-32211, LI-COR Biosciences). Immunoreactive bands were detected using an IR-Odyssey scanner (LI-COR Biosciences). The ratio of pVASP/VASP was calculated using Image Studio Lite Version 3.1.

To further localize the expression of pVASP and determine the role of cGMP, additional hearts were collected for immunofluorescence using a protocol described elsewhere (37). Briefly, the hearts were given the supernatant of RBCs or exogenous cGMP and subsequently subjected to IR as above. After collection, the hearts were perfused with 4% formaldehyde and fixed for 24 hours at room temperature, dehydrated in graded ethanol (70, 95, and 99%), embedded in paraffin, sectioned using a microtome, and mounted on coated glass slides (Superfrost plus; Thermo Fisher Scientific). At least 6 slides containing approximately 4 tissue cross sections in a thickness of 5 μm from each heart were examined. Sections were deparaffinized in xylene and rehydrated in graded ethanol. For antigen retrieval, slides were merged in a high-pressure boiling citrate buffer (pH 6.0). Heart cross sections were permeabilized with 0.3% Triton X-100 for 10 minutes, blockade with goat serum (Abcam), and incubated overnight (4°C) with the following primary antibodies: a rabbit polyclonal anti-pVASP (phosphorylation at Ser239; 1:100 dilution, IgG, catalog PA5-99,377; Thermo Fisher Scientific) and a mouse monoclonal anti-myosin heavy chain 7 (MYH7; 1:100 dilution, IgG_2b_, catalog MABT838; Sigma-Aldrich). Specific labeling was detected with an Alexa Fluor 488 goat anti-rabbit (1:200 dilution, Life Technologies) or an Alexa Fluor 594 goat anti-mouse (1:200 dilution, Life Technologies), respectively. Cell nuclei were counterstained with Hoechst dye (Sigma-Aldrich). To confirm the specificity of antibodies, isotype controls were used as negative controls (rabbit IgG or mouse IgG, Abcam). Fields were captured with the fluorescence microscope equipped with an ×40 objective lens and an ×10 eyepiece (Leica DM3000 digital microscope; Leica Biosystems), digitized, and analyzed (ImageJ software 1.53v).

### Cyclic GMP detection in supernatant of RBCs.

Mouse RBC suspension (hematocrit about 70%) was exposed to hypoxia (1% O_2_ and 5% CO_2_) or normoxia (21% O_2_ and 5% CO_2_) in the presence of the PDE5 inhibitor sildenafil (10 mmol/L, Sigma-Aldrich) at 37°C for 1 hour. The supernatant was collected following centrifugation at 1,000*g* and 4°C for 5 minutes. cGMP in the supernatant was determined by ELISA using the cGMP complete kit (ADI-900-164; Enzo) according to the manufacturer’s instructions. The 96 microplate was read at 405 nm using a Victor Multilabel Plate Reader (2030-0050; PerkinElmer).

### Statistics.

LVDP during reperfusion is expressed as a percentage of the preischemic values. The differences in functional cardiac parameters were analyzed by 2-way ANOVA followed by Tukey’s multiple comparison test. Differences in infarct size were analyzed using 1-way ANOVA followed by Tukey’s multiple comparison test or Kruskal-Wallis test followed by Dunn’s multiple comparison test, depending on the distribution of data. Differences between 2 groups were analyzed by unpaired or paired 2-tailed *t* tests, Wilcoxon or Mann-Whitney test, depending on the distribution. Normality was checked with D’Agostino Pearson’s test or Shapiro-Wilk test, depending on the number of observations in each group. All statistical analysis was performed using Prism Version 7.04 (GraphPad Software). Unless otherwise stated, data are presented as mean ± SD, and *P* < 0.05 was considered statistically significant.

### Study approval.

All animal experiment protocols were approved by the Ethical Committee of Stockholm and conform to the Guide for the Care and Use of Laboratory Animals published by the United States National Institute of Health (NIH publication No. 85-23, revised 1996). All procedures involving humans were conducted according to the Declaration of Helsinki. The protocol was approved by the Swedish Ethical Review Authority for human studies. All participants were informed of the study’s purpose and gave their oral and written informed consent before any study-related procedures were initiated.

### Data availability.

Supporting data values for all data presented as means are available in the Supplemental Data Values file. Data can also be accessed from the corresponding author upon request.

## Author contributions

JY, EW, JOL, and JP conceived and designed the study. JY and TJ performed and collected research data. JY and JP analyzed research data and performed statistical analysis. MLS, EW, JOL, and JP recruited patients and collected samples. JY, JOL, and JP wrote the manuscript. XZ, TJ, AC, YT, AM, JT, EM, SBC, ZZ, MMCK, TA, MC, and EW edited the manuscript, and all authors reviewed and approved the final version of the manuscript.

## Supplementary Material

Supplemental data

Supporting data values

## Version 1 09/01/2023

Electronic publication

## Version 2 09/06/2023

Term clarification on page 7
