# Supplementary material for: Hypoxic erythrocytes mediate cardioprotection through activation of soluble guanylate cyclase and release of cyclic GMP
Source: J Clin Invest. 2023 Sep 1;133(17):e167693. doi: 10.1172/JCI167693 (PMC10471167; doi:10.1172/JCI167693)
Supplement: Supplemental data [file jci-133-167693-s010.pdf]

Supplemental figures and figure legends

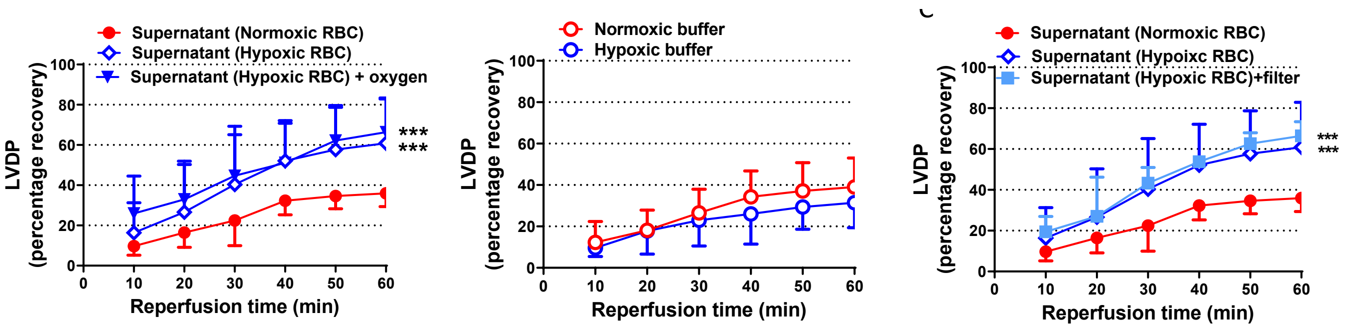

Supplemental Figure 1. Red blood cells (RBCs) release a cardioprotective factor during hypoxia. Recovery of LVDP during reperfusion following administration of (A) supernatant from normoxic RBCs (n=5), supernatant from hypoxic RBCs after reoxygenation in 21% O_2 (n=7) and buffer exposed to hypoxia (n=8), (B) normoxic or hypoxic buffer (n=8) and (C) supernatant from normoxic RBCs (n=5), hypoxic RBCs (n=11) and hypoxic RBCs after passage through a 100 kDa filter (n=4). The recovery of LVDP is expressed as percentage of the pre-ischemic level. Data are presented as mean ± SD. *** $P<0.001$ denotes significant differences to normoxia by two-way ANOVA.

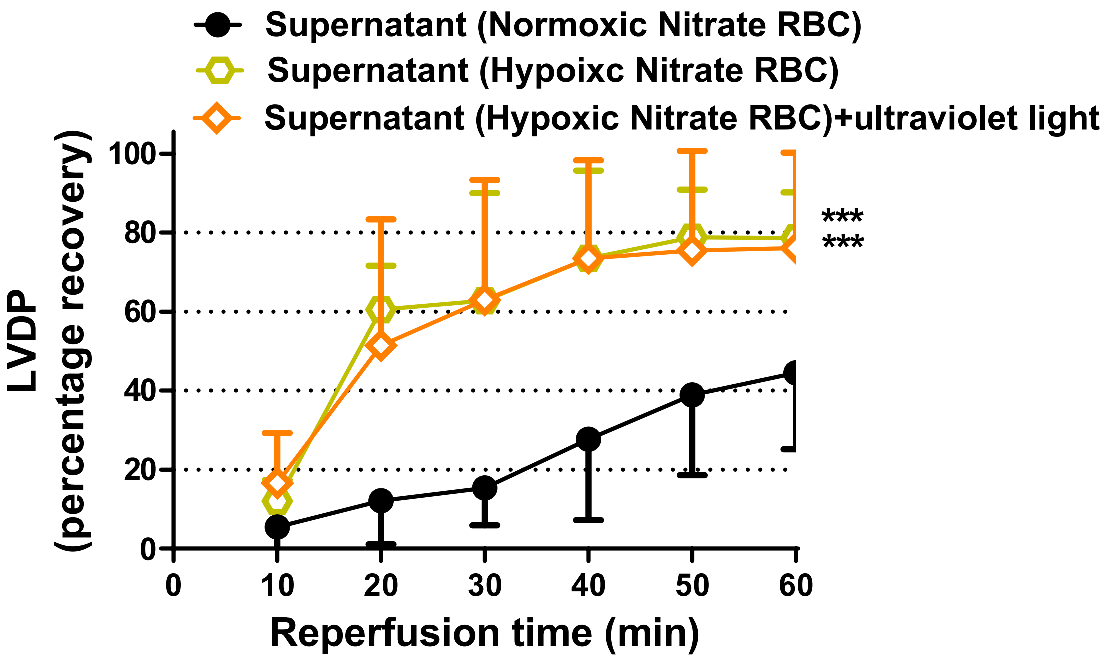

Supplemental Figure 2. Ultraviolet light does not block protective effect by hypoxic RBCs. Recovery of LVDP during reperfusion following administration of supernatant from normoxic RBCs from nitrate treated-mice (n=5), hypoxic RBCs (n=5) and hypoxic RBCs after exposure of supernatant to ultraviolet light (n=5), Data are presented as mean ± SD. ***p<0.001 denotes significant differences to normoxic RBCs by two-way ANOVA.

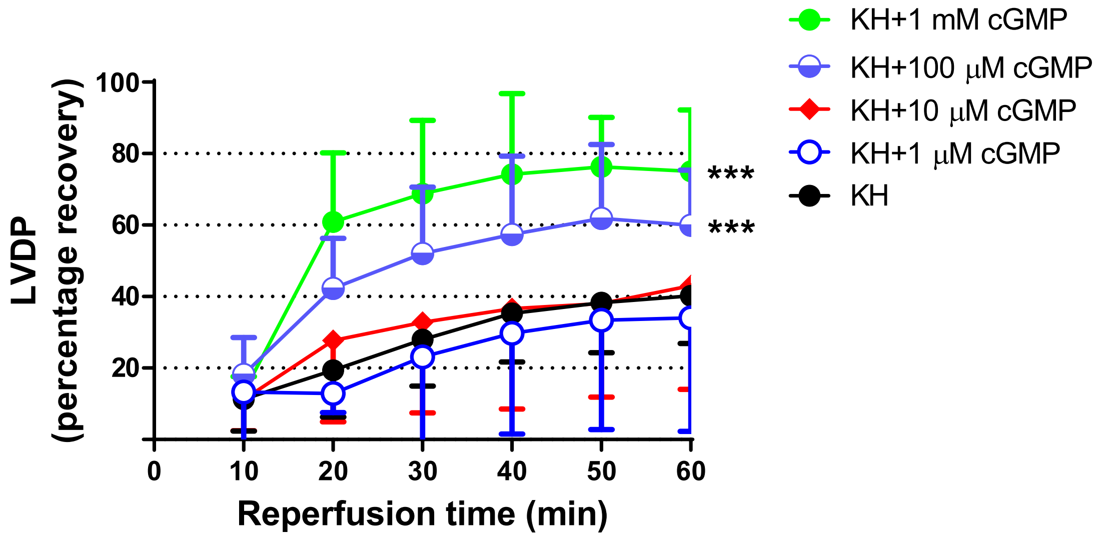

Supplemental Figure 3. Exogenous cGMP induces cardioprotection. Recovery of left ventricular developed pressure of isolated hearts given increasing concentrations of cGMP in Krebs-Henseleit (KH) buffer. ***P<0.001 denotes significant differences to KH using two-way ANOVA, n=4-6.

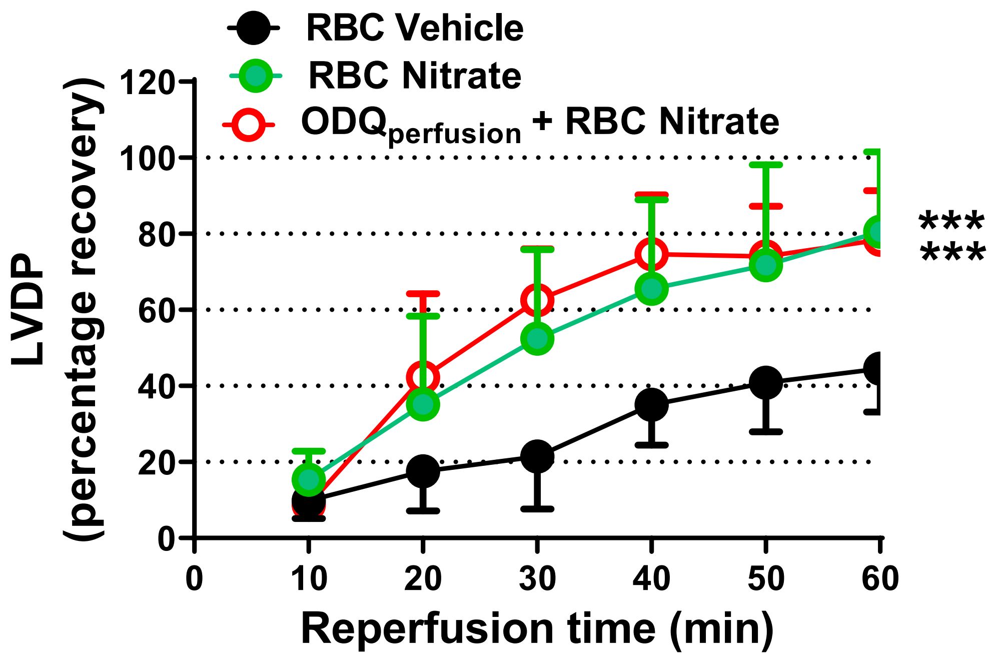

Supplemental Figure 4. Inhbition of sGC in the heart does not affect the cardioprotective effects of RBCs. Recovery of LVDP during reperfusion following administration of RBCs from mice given normal drinking water (Vehicle, n=7)) or nitrate (n=9) under control conditions or following perfusion of the heart with the sGC inhibitor ODQ (n=5). Data are presented as mean ± SD. ***P<0.001 denotes significant differences to the vehicle group by two-way ANOVA.

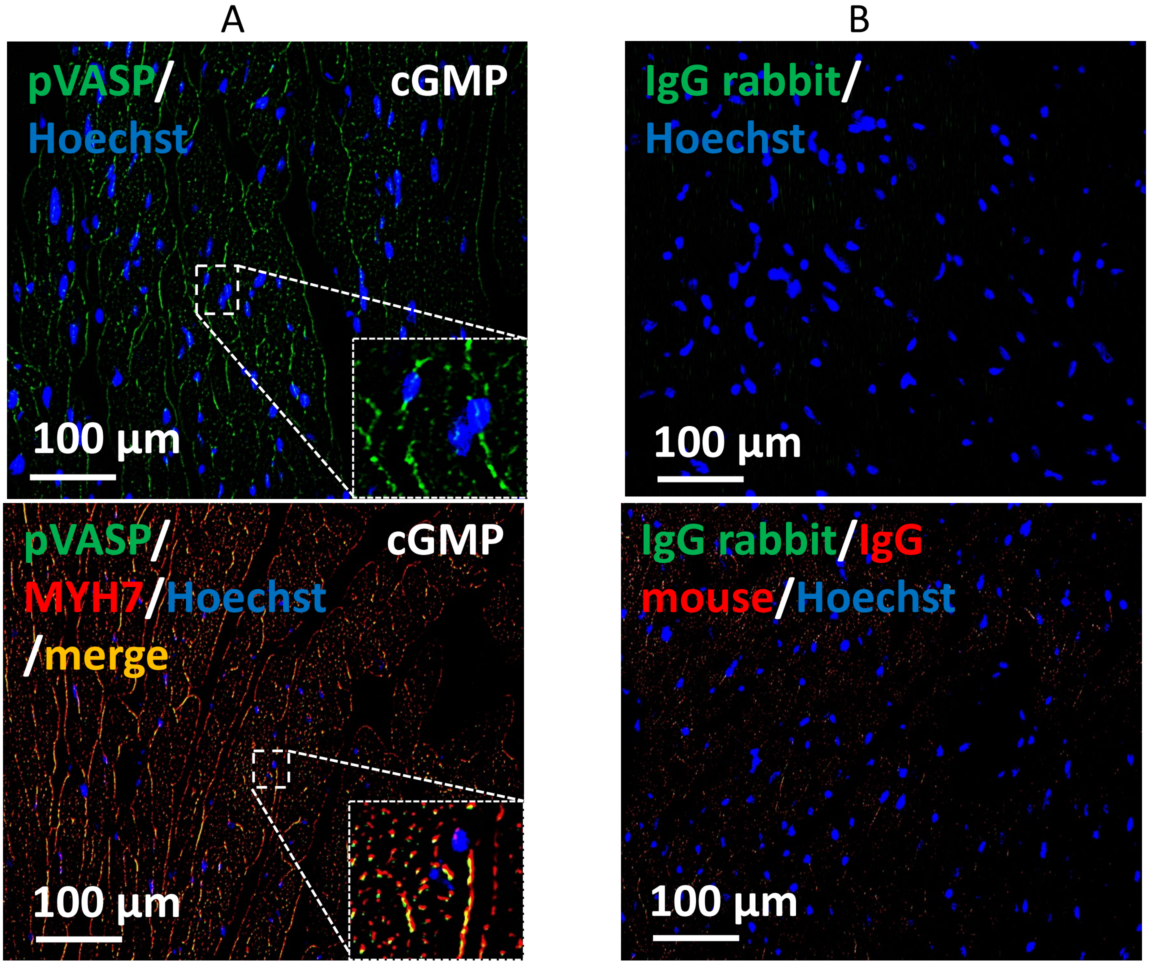

Supplemental Figure 5. Positive (A) and negative (B) immunofluorescence staining in cardiac tissue. Positive staining was induced by exogenous cGMP (100 mM) in the presence of pVASP was visualized using Alex FluorTM 488 antibody (green) and the cardiomyocyte specific marker myosin heavy chain 7 (MYH7, red). Nuclei were stained with Hoechst (blue). Negative staining was induced by rabbit and mouse IgG following administration.

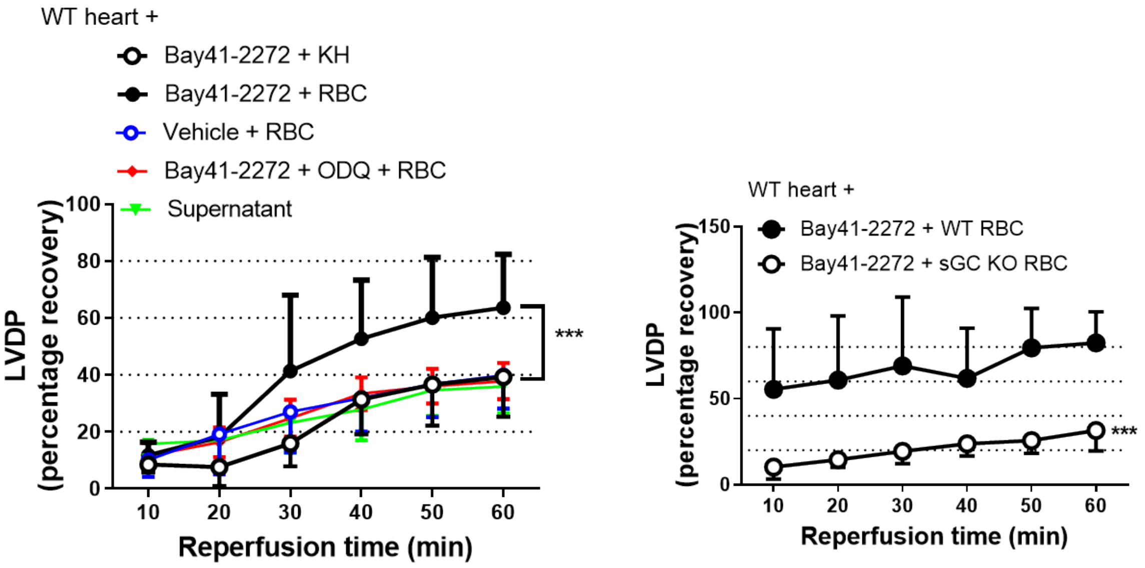

Supplemental Figure 6. Pharmacological stimulation of sGC in RBCs protects the heart. Recovery of left ventricular developed pressure of isolated hearts given the sGC stimulator Bay 41-2272 in the presence of Krebs-Henseleit (KH) buffer or RBCs (Left). Bay41-2272 improved cardiac recovery in the presence of RBCs. This effect is blocked by the sGC inhibitor ODQ. The protective effect is also absent when using RBCs from sGC knockout mice (Right). ***P<0.001 denotes significant differences to the vehicle group or wild-type (WT) RBCs by two-way ANOVA, n=4-6.

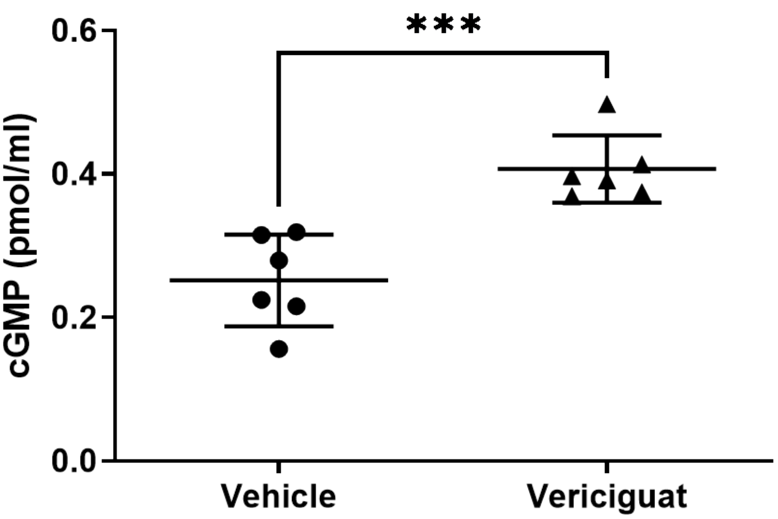

Supplemental Figure 7. Pharmacological sGC stimulation in RBCs induce release of cGMP. cGMP levels in the supernatant collected from RBCs incubated with vehicle or the sGC stimulator vericiguat. ***P<0.001 denotes significant by unpaired t-test.
